# Valley network morphology in the greater Meridiani Planum region, Mars

**DOI:** 10.1080/17445647.2018.1530154

**Published:** 2018-11-15

**Authors:** Frank C. Chuang, Rebecca M. E. Williams

**Affiliations:** Planetary Science Institute, Tucson, USA

**Keywords:** Valley networks, Meridiani Planum, geomorphology, groundwater, Mars

## Abstract

The Greater Meridiani Planum region on Mars is a key locale for a diverse range of fluvial landforms. Valley networks in this region have a range of geomorphologic styles that include negative relief, positive relief, or some combination of both along their lengths. Using high-resolution ~5–6 m/pixel orbital images in ArcGIS Desktop software, we mapped previously under-recognized fine-scale valley networks within the Greater Meridiani Planum region and recorded their geomorphic characteristics as feature attributes. The objectives in using the mapped features are to 1) document the full range of valley network morphologic types in the region, 2) document changes in morphologic types both on a regional scale and along the valley network segments, and 3) to use the mapped features along with other geologic information from previous studies to better understand landscape evolution in the Greater Meridiani Planum region.

## 1. Introduction

The relative timing and duration of aqueous events on Mars is of great interest in identifying former habitable environments. Valley networks are the most common drainage feature on the planet and they are frequently cited as evidence of former clement climate conditions (e.g. Carr & Head, 2010). ‘Classic’ valley networks, identified in early Viking images of Mars, are typically negative relief branching features that are a few kilometers wide, >100 m deep, and up to 200 km long (e.g. Mars Channel Working Group, 1983). Recent studies have also recognized a range of preservation states for valley networks (Williams, Irwin, Burr, Harrison, & McClelland, 2013, 2017) including sinuous ridges formed by landscape inversion that demarcate former fluvial pathways (often termed ‘inverted channels’; e.g. Burr et al., 2009; Pain, Clarke, & Thomas, 2007). Documenting the spatial distribution of both ‘classic’ valley networks (Hynek, Beach, & Hoke, 2010) and other preserved morphological types expands our understanding of where and when surface aqueous conditions occurred in Martian history.

**Figure 1.**
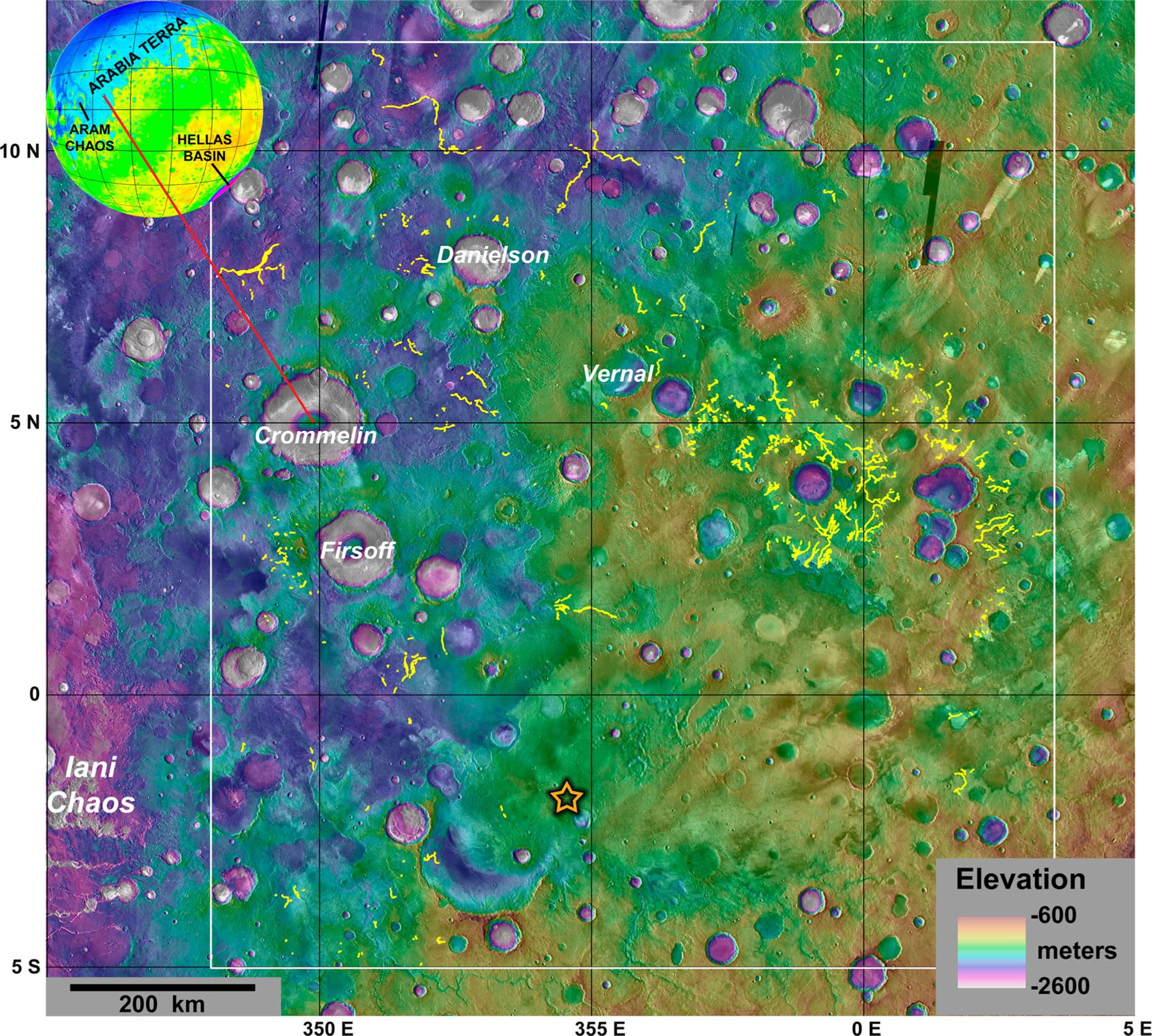
Location of the GMP region on Mars near the southwestern edge of the more extensive Arabia Terra terrain. The white box denotes the mapping area and the yellow lines are fine-scale valley networks mapped in this study. The orange star outlined in black is the landing site of the MER Opportunity rover. Background consists of colorized global MOLA gridded elevation data (~463 m/pixel) draped over global THEMIS daytime IR data (~100 m/pixel).

The vast Arabia Terra region of Mars has numerous inverted valley network systems that have been recently mapped by Davis, Balme, Grindrod, Williams, and Gupta (2016). This study is focused on under-recognized fine-scale valley networks in a smaller portion of Arabia Terra between 5°S–12°N and 348°E–3.5°E, often referred to as Greater Meridiani Planum (GMP). The GMP region covers parts of four 1:5,000,000 scale Mars quadrangles including Oxia Palus (Mars Chart 11; 0–30°N, 315–360°E), Arabia (Mars Chart 12; 0–30°N, 0–45°E), Margaritifer Sinus (Mars Chart 19; 0–30°S, 315–360°E), and Sinus Sabaeus (Mars Chart 20; 0–30°S, 0–45°E). Our mapping of GMP valley networks (Main Map), which have both negative and positive relief, have revealed additional details about the aqueous history of the region (Figure 1). The diverse stratigraphic, morphologic and planimetric attributes enable a refined spatial and temporal reconstruction of the ancient aqueous history in the GMP region not previously obtainable due to the lack of high-resolution images and detailed geology.

## 2. Data and methods

### 2.1. Data types and sources

The primary data set for mapping valley networks in this study is Mars Reconnaissance Orbiter (MRO) ConTexT (CTX; Malin et al., 2007) images at ~5–6 m/pixel resolution. Where available, higher resolution images from the MRO High-Resolution Imaging Science Experiment (HiRISE, ~0.25 m/pixel maximum; McEwen et al., 2010) and the Mars Global Surveyor (MGS) Mars Orbiter Camera (MOC, ~0.5 m/pixel maximum; Malin & Edgett, 2001) were used for additional geomorphologic investigation. The thermal characteristics (infrared spectrum of light) of the surface from global day-time and night-time Thermal Emission Imaging System (THEMIS; ~100 m/pixel; Christensen et al., 2004) data were used to help delineate geologic units mapped at 1:2,000,000 scale by Hynek and Di Achille (2017) in the GMP region. The THEMIS data, which is geodetically-controlled to the most recent geoid for Mars, served as the basemap for the map region. Image data obtained for this study are typically available as map-projected JPEG2000 (.jp2), GeoTiff (.tif) or cube (.cub) files. The latter is a file format specific to Integrated Software for Imaging Spectrometers (ISIS; http://isis.astrogeology.usgs.gov) software used by the planetary science community. Global topographic data from the MGS Mars Orbiter Laser Altimeter (MOLA; ~463 m/pixel; Smith et al., 2001) instrument was also used to estimate the elevation differences of geologic units in the study region. The geologic map of the GMP region, available as an ArcGIS geodatabase package and individual shapefiles, was acquired through the United States Geological Survey (USGS) Publications Warehouse (https://pubs.er.usgs.gov).

The images used in this study are in the public domain and were obtained from websites that are affiliated with the National Aeronautics and Space Administration (NASA) Planetary Data System (https:// http://pds-geosciences.wustl.edu) or home institutions of individual spacecraft instruments. These include the Mars Orbital Data Explorer (http://ode.rsl.wustl.edu/mars), the Mars Odyssey THEMIS instrument (https://themis.asu.edu), and the USGS Planetary Image Locator Tool (https://pilot.wr.usgs.gov).

### 2.2. Software and mapping methods

All data in this study were integrated into the Environmental Systems Research Institute’s ArcGIS Desktop 10.4 (ArcGIS) software and co-registered to the global THEMIS basemap. Projection of the basemap is defined using the International Astronomical Union Mars 2000 geoid for Mars (Seidelmann et al., 2002). Data used in this study is typically in simple cylindrical or equirectangular map projection, but for the purposes of accuracy in area and length measurements, they were re-projected to a sinusoidal map projection with a center longitude of 358°E.

CTX images are released to the public used an elliptical geoid of Mars that is different from most other released digital data for Mars (i.e. spherical). This causes offsets by as much as several hundred meters both longitudinally and latitudinally relative to image data that is controlled to the THEMIS basemap. Although the offsets can be greatly minimized by either manually shifting or ‘rubber sheeting’ the images using ‘tie’ points in ArcGIS, the co-registration was not 100% accurate. However, this did not compromise our ability to map fine-scale valley networks using CTX images, the primary dataset for this study.

Using ArcGIS, we initially located and studied the overall patterns of valley networks in CTX images at various scales up to 1:50,000. This helped provide context for our mapping and to differentiate valley network types from other non-fluvial landforms. After the initial survey, we then manually digitized individual valley network segments as line features at a consistent scale of 1:1,000. The morphological information for each feature were saved as text or numerical attributes. Included in these attributes are the geologic unit(s) from Hynek and Di Achille (2017) that an individual valley network segment is present within. We note that any age associated with a valley network based on the map units should be taken in the broadest possible terms as there could be variations in the local geology at CTX scales that are not detectable in the THEMIS-scale geologic map. Other manually digitized features in this study include polygons of candidate paleolakes observed at the ends of several valley networks. All mapped features are stored in an ArcGIS file geodatabase (see supplemental materials) and if necessary, output as shapefiles which is a standard industry format for GIS.

## 3. Valley networks

### 3.1. Morphologies

**Figure 2.**
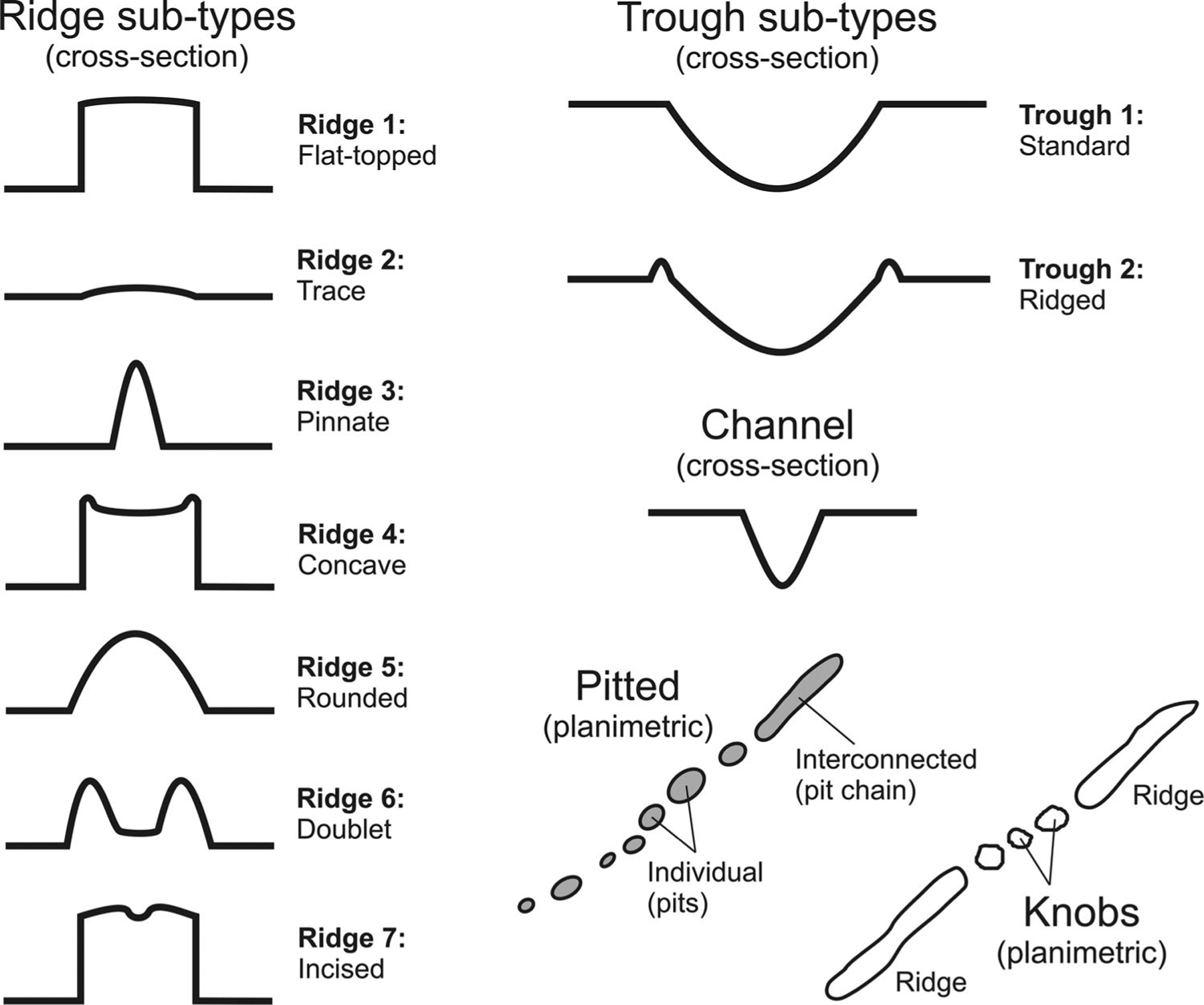
Schematic illustration of the cross-sectional and planimetric shapes for the various principle types and sub-types of mapped fine-scale valley networks. Note that the illustrations are idealized. Each of the types have either negative or positive relief. Negative forms include channels, troughs, and pits. Positive forms include ridges and knobs. See text for details regarding each type. Scale-wise, troughs are much wider features than channels and planimetric features such as pits and knobs are smaller than ridges, troughs, and channels.

From our mapping, fine-scale valley network morphologies can be classified into four principle types: ridges, channels, pitted and knobs. An additional classified type are troughs, which are generally less common than the four principle types. Within each principle type, there can be slight variations of the main feature and thus, several sub-types. All of the principle and sub-types are stored as attributes. Descriptions of each type are below with the ArcGIS attribute text designation given in parentheses, and cross-sectional illustrations of the types in Figure 2.

#### 3.1.1. Ridges

**Figure 3.**
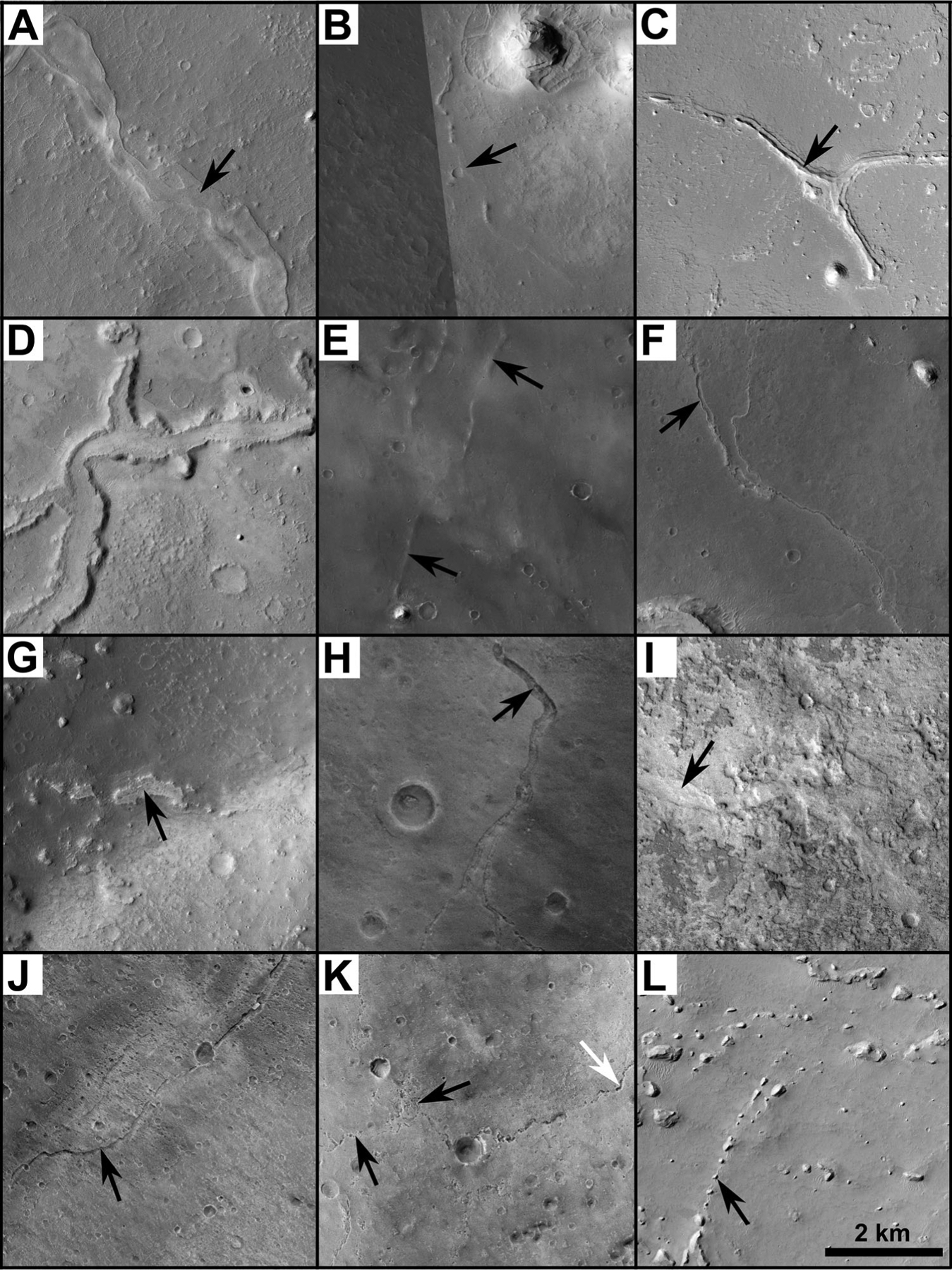
Examples of the principle morphologic types and sub-types for fine-scale valley networks mapped in this study. Ridge sub-types include: (A) flat-topped, (B) trace, (C) pinnate, (D) concave, (E) rounded, (F) doublet, and (G) incised. Trough sub-types include: (H) standard and (I) ridged. Other principle types include (J) channels, (K) pits, and (L) knobs. Arrows in each panel point to the features of interest. White arrow in panel K points to interconnected pits that have formed a pit chain. See text for more details regarding each type and sub-type. The scale is the same in each of the panels (bar in lower right of panel L) and sun illumination is from the left. The approximate center location of the CTX image shown in each panel are: (A) 352.6°E, 5.5°N; B03_010697_1875, (B) 353.0°E, 5.7°N; B05_011409_1837, (C) 355.9°E, 7.1°N; B10_013611_1894, (D) 354.6°E, 9.4°N; F03_036898_1891, (E) 359.9°E, 2.9°N; P03_002113_1825, (F) 357.7°E, 4.2°N; F03_036977_1856 and G09_021905_1848, (G) 352.1°E, 7.9°N; B03_010697_1875, (H) 358.8°E, 4.5°N; P11_005436_1867, (I) 351.9°E, 6.3°N; P07_003933_1862, (J) 0.1°E, 3.9°N; P13_005937_1834, (K) 359.9°E, 3.1°N; P03_002113_1825, and (L) 354.6°E, 1.6°N; P20_008785_1836.

Ridges are 20–900 m wide positive-relief features that are mostly flat-topped or rounded in cross-section (Figure 3(A–G)). Overall, seven sub-types are defined: flat-topped (ridge1), trace (ridge2), pinnate (ridge3), concave (ridge4), rounded (ridge5), doublet (ridge6), and incised (ridge7). Trace ridges are cases in which the ridge form is largely absent, but is faintly detectable from shading or shadows. Concave is where narrow sharp ridges bound the sides of the main ridge with a central convex-up appearance. Doublet ridges are cases where two conformal ridges make up the overall ridge form, analogous to the shape of a terrestrial leveed river valley. Incised ridges are where a single narrow channel incises the central portion of the ridge top. All other types describe the main ridge form.

#### 3.1.2. Troughs

Troughs are negative-relief, convex-up features that are generally >100 m wide along their paths (Figure 3(H and I)). Two sub-types are observed: standard (trough1) and ridged (trough2).

#### 3.1.3. Channels

Channels (channel) are linear to sinuous negative-relief features < 100 m long (Figure 3(J)). Their cross-sectional forms range from shallow-wide to deep-narrow with a flat or convex floor.

#### 3.1.4. Pitted

Pitted types (pits) consist of aligned circular to oval shaped features with the short axis ranging from 10 m to 100 m (Figure 3(K)). Pits can be singular features, but often combine and interconnect with adjacent pits to form pit chains.

#### 3.1.5. Knobs

Knobs (knob) are hills with flat or rounded tops found in association with ridges (Figure 3(L)). Their sizes can vary, but are generally not wider than the original ridge feature.

### 3.2. Other attributes

In addition to morphologic type, we also include four other attributes of mapped valley networks based on 1) their grouping as part of a larger overall networked system, 2) their location within Hynek and Di Achille (2017) geologic map units, 3) their stream order in the Strahler ordering system (Strahler, 1952), and 4) their drainage pattern based on classic terrestrial patterns (Morisawa, 1985; Summerfield, 1991). For groupings, valley network segments are given an identification number as part of a uniquely-defined networked system. In this study, Strahler network order numbers range from one to four. Drainage patterns for valley networks have a single or combination of two patterns that include dendritic, rectilinear, parallel, trellis, rectangular, dendritic-rectilinear, rectilinear-parallel, trellis-parallel, trellis-rectilinear, and none.

## 4. Results and discussion

Previous studies of Martian valley networks have recognized the diversity of morphologic types in the GMP region (e.g. Edgett, 2005; Davis et al., 2016). In this study, we have mapped and documented fine-scale valley networks that specifically have pitted morphologies, transitions between two or more morphologic types, and concentrations of morphologic types within exposed ancient cratered terrain. Overall, there appears to be a major southeast to northwest change in valley network morphologic type, density, and planimetric pattern that is consistent with the regional down-sloping topographic gradient.

We have mapped a total of 4,629 individual valley network segments and 190 unique groupings within the study region (see formal map). The total length of all mapped segments is 5,143.1 km. Of these, approximately two-thirds (67%; sum length = 3,443.6 km) are within geologic unit *Nhc1*, interpreted by Hynek and Di Achille (2017) as exposed ancient cratered terrain, the lowermost unit in the GMP region. The remaining one-third of mapped valley network segments are primarily within unit *Nhc2*, a younger Noachian-aged cratered terrain, and various ‘etched’ units (*NMe1*, *NMe2*, *HNMe3*) that formed during the Noachain-Hesperian transition period. The etched units consist of degraded layered deposits that once mantled much of the GMP region, but are now less extensive and provide windows down into ancient cratered terrain (Edgett, 2005; Edgett & Malin, 2002; Hynek & Di Achille, 2017; Zabrusky, Andrews-Hanna, & Wiseman, 2012). Several of the single-threaded ridge forms within etched units cross into ancient cratered terrain, which shows the correspondence of these two units and thus, the erosional nature of the etched units. Few valley network segments are mapped in the stratigraphically highest Hesperian plains units (*Hp* or *HMh*).

Transitions between morphological types along course were observed in ~14% of all valley network segments. Most segments within larger networked systems had at least two different morphologies (e.g. knob-ridge, pit-channel, ridge-channel, etc.) and in some cases, as many as three. We recorded 54 unique transitions between two or more morphological types along all mapped segments and these are displayed on the formal map with a common line color (white). Nearly half (42%) of transitional types are pit-channel varieties.

**Figure 4.**
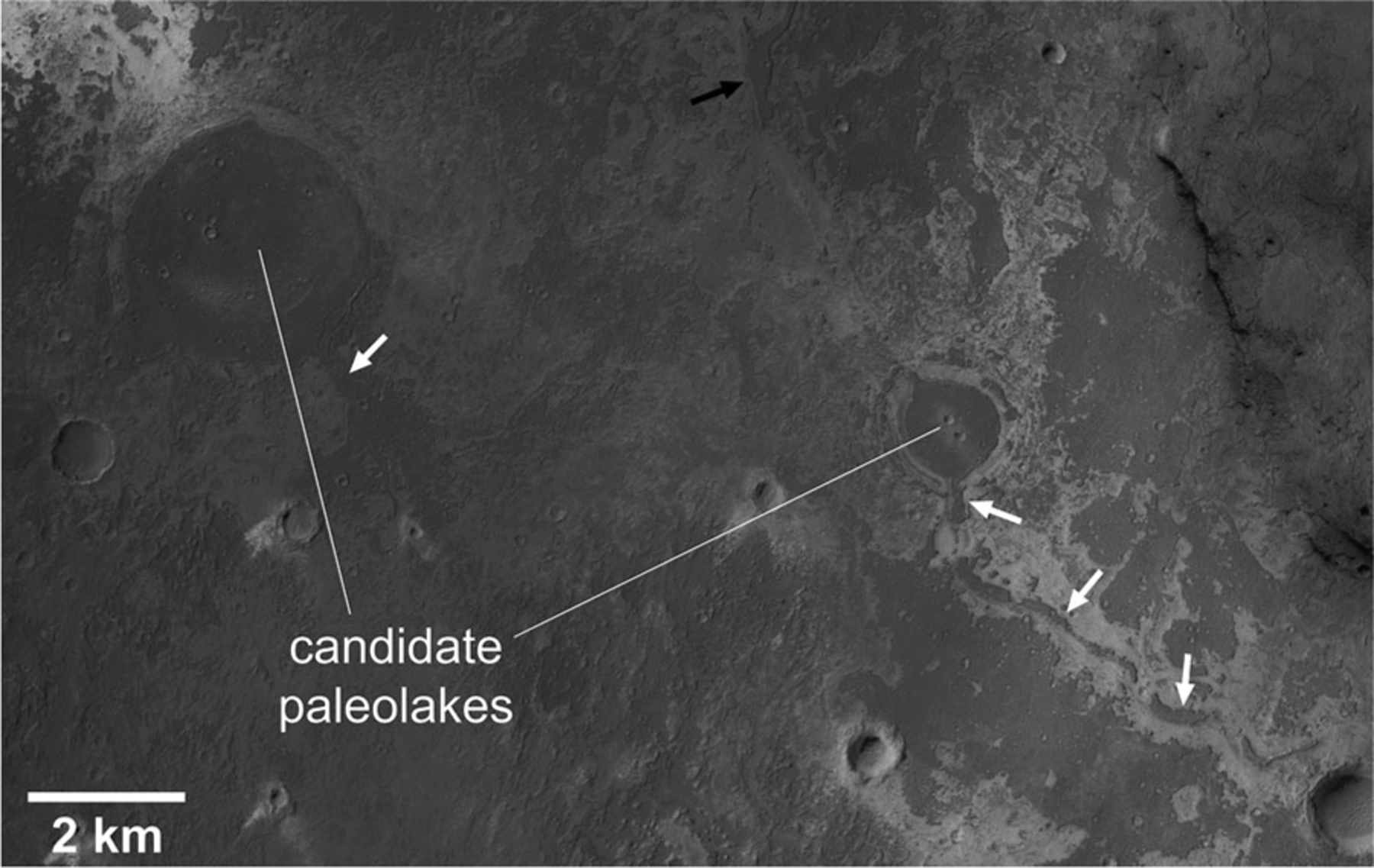
Example of candidate paleolakes within the western exposure of *Nhc1* ancient cratered terrain (see Figure 5 for context). Dark-toned ridge type valley network segments (white arrows) lead into a large circular plateau are potentially remnant deposits that once filled channels and craters, but are now high-standing features due to differential erosion. The circular plateaus may have been former impact craters that were breached by valley networks and then filled with lakebed deposits. For the paleolake on the right, continuation of a ridge to the northwest (black arrow) indicates that the crater rim may have been breached, forming an outlet for the paleolake. Portion of CTX image B16_015932_1884 centered at ~351.6°E, 6.4°N. Sun illumination is from the left.

At the apparent terminus of some ridges are a single large, circular-to-sub-circular raised plateau interpreted to be a candidate paleolake (see Davis et al., 2016; Figure 4). A total of fifteen paleolakes were mapped. These features occur in both the ancient cratered and etched geologic units throughout the map region. They are also more common in the northern half of the map which is lower in elevation than the southern half.

### 4.1. Valley networks in ancient cratered terrain and etched units

**Figure 5.**
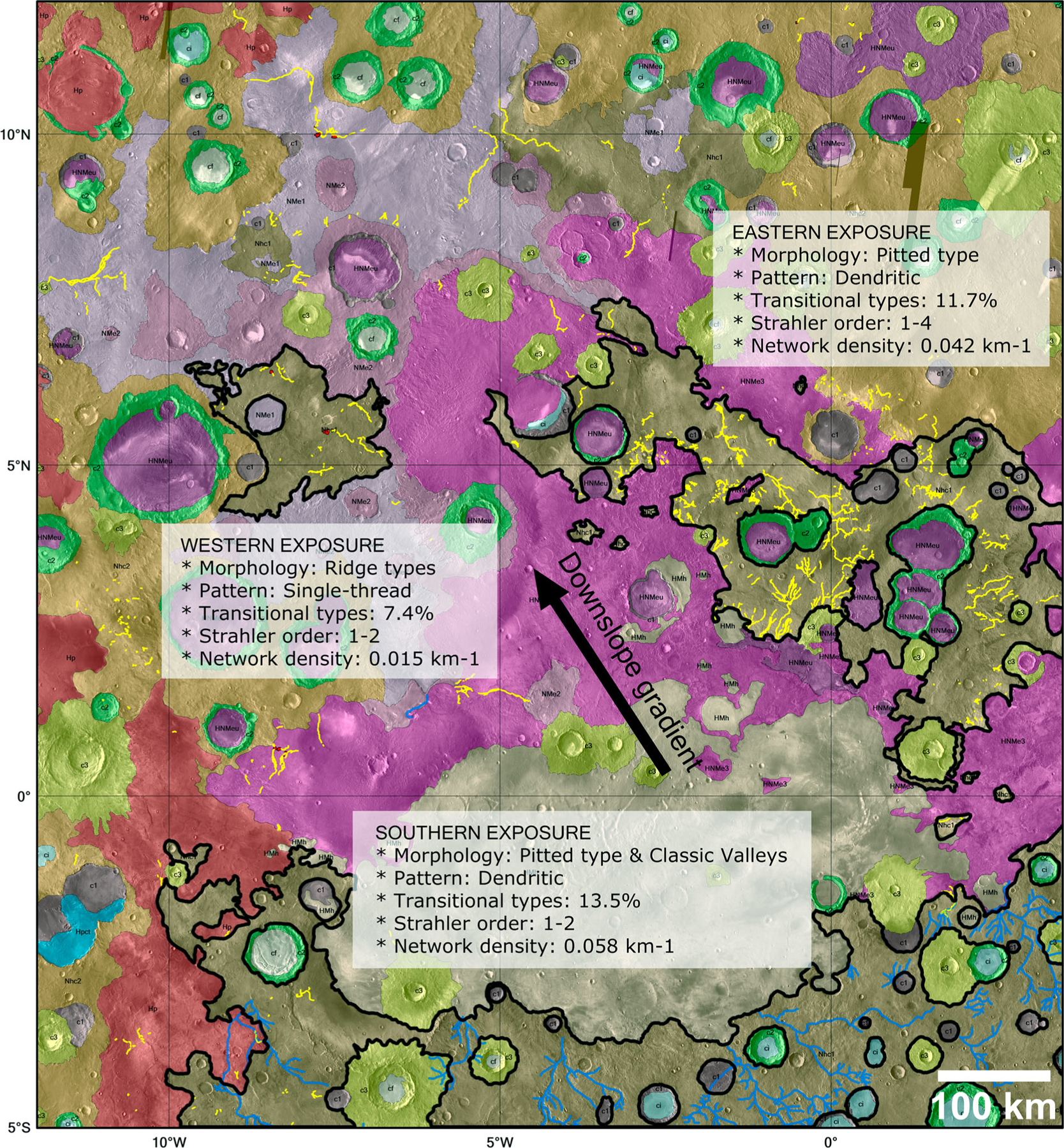
Three large regional exposures (southern, eastern and western) of geologic unit *Nhc1* (black outline) from Hynek and Di Achille (2017) highlighted in the map region with mapped fine-scale valley networks (yellow lines). Valley network characteristics of each exposure are listed in the white boxes. For more details, see section 4 of the text. The downslope gradient in the map region (black arrow) is towards the northwest, where most candidate paleolakes (red areas outlined in black) are located. Background consists of the colorized geologic map by Hynek and Di Achille (2017) draped over global THEMIS daytime IR data (~100 m/pixel).

The morphology (singular and transitional types), drainage pattern, and network density of fine-scale valley networks differ markedly across the region (Figure 5). The latter is simply the total length of all valley network segments divided by the area of the *Nhc1* exposure (see Williams, Chuang, & Berman, 2017). The regional differences in valley network morphology are especially apparent when comparing locations of unit *Nhc1*, where the majority of fine-scale valley networks in GMP are located. Exposures of *Nhc1* occur in three large areas: western, eastern, and southern (see Figure 5).

The southern exposure has the fewest fine-scale networks among the three areas. Of the 96 mapped segments, about 71.9% (69/96) are pitted types. This area instead is dominated by much larger, classical-style, dendritic valley networks that have been mapped in detail by Hynek et al. (2010) and in earlier studies using Viking Orbiter mission data (Baker, 1982; Carr, 1981; Carr, 1996; Pieri, 1980 and references therein). Among classic valley networks on Mars, this particular southern exposure has the highest drainage density (0.14 km^−1^) and Strahler stream order (7) on the planet (Hynek et al., 2010); and compared to fine-scale valley networks in the other two exposures, has the highest network density (0.058 km^−1^) among the three.

Proceeding down gradient to the eastern exposure, this area has an abundance of dendritic fine-scale networks that are dominantly pitted (77.9%, 2539/3258). The pits are often aligned and interconnected, forming chains in overall branching patterns. Williams et al. (2017) interpreted the pits as dissolution features along relict buried stream courses. This area has a slightly lower network density of 0.042 km^−1^. Many of the networks can be traced to localized source regions, often located at the base of scarps along the contacts between etched and ancient cratered terrains as well as crater units. Further down gradient to the west, the western exposure has mostly single-thread ridge types (61.1%). Fine-scale valley networks in this exposure have little branching, low stream order (up to 2), and a network density of 0.015 km^−1^. These regional morphologic differences indicate a changing regime of aqueous activity over time and geographic space for two-thirds of the mapped valley networks.

The remaining one-third of fine-scale valley networks have characteristics that are also in line with the aforementioned regional changes. Unit *Nhc2*, interpreted by Hynek and Di Achille (2017) as a slightly younger ancient cratered terrain that has retained more impact craters than *Nhc1*, has 410 mapped segments that cover a total length of ~302.4 km. The dominant morphologic type in *Nhc2* is pitted with a very similar percentage to the eastern *Nhc1* exposure, ~74.1% (304/410). This result is consistent with the interpretation that *Nhc2* is nearly coeval with *Nhc1* and are of the same ancient cratered material (Hynek & Di Achille, 2017).

Etched units *NMe1*, *NMe2*, and *HNMe3* have a total of 504 mapped segments covering a total length of ~1120.6 km. Of these, ~60.3% are ridge types (304/504). If transitional types with at least one ridge type are included, then this increases the percentage of segments with ridge types to ~67.9% (342/504). Strahler stream orders among the 342 ridge segments are no higher than two with the vast majority (87.7%; 300/342) having an order of one. This low stream order indicates mainly single-thread ridge types, consistent with the western exposure of *Nhc1*. Furthermore, as all etched units are stratigraphically younger than ancient cratered terrain, it suggests that single channel flow near the end of the regional transition (south --> east --> west) for ancient cratered terrain may have continued into the period of etched unit formation.

### 4.2. Landscape evolution of the GMP mapping region

Here, we briefly summarize the nature and relative timing of the fine-scale valley networks in this study that are discussed in greater detail by Williams et al. (2017), including the use of terrestrial analogs and the geologic and hydrologic histories of the GMP region (Davis et al., 2016; Edgett, 2005; Hynek & Di Achille, 2017). Prior to the Hesperian, dendritic valley network formation may have been common in the eastern and southern portions of the map region with aqueous flows that traveled along shallow gradients towards the north-northwest into a local sedimentary sink. Shallow lakes formed in this part of the map region as evidenced by the number of mapped paleolakes. Near the Noachian-Hesperian transition (~3.7 Ga), burial of the GMP region by layered sedimentary materials formed the etched units with fluctuating water tables resulting in sulfate precipitation (Andrews-Hanna, Zuber, Arvidson, & Wiseman, 2010; Wiseman et al., 2010). Subsequent erosion then stripped away much of the etched units by ~3.5 Ga (Zabrusky et al., 2012) with groundwater dissolution likely involved in liberating sulfate-cemented materials for transport.

Regional differences in valley network characteristics may be related to the mobility of solutes, reflecting temporal changes in aqueous processes. Surface deflation likely resulted in the formation of exhumed fluvial systems (ridges) in western *Nhc1*, *Nhc2*, and the etched units whereas the pitted valley network systems with transitional curvilinear depressions and ridges (in eastern *Nhc1* and parts of *Nhc2*) are likely due to reactivation of former valley network conduits from groundwater-fed dissolution/precipitation processes following widespread erosion of the etched units. Thus, the central GMP region may have once been a site of abundant groundwater activity on Mars. Overall, these results are indicative of surface conditions that were warm enough to support liquid water at times during the Hesperian or later.

## 5. Conclusions

Our mapping of fine-scale Martian valley networks at CTX-scale has demonstrated that the majority of these features (~67%) are associated with the lowest stratigraphic units in the GMP region and, more specifically, in ancient cratered terrain. Three regional exposures of this terrain (*Nhc1*) are apparent with each having valley network characteristics that differ from one another. The southern exposure is dominated by classical-style dendritic valley networks with a network density of 0.058 km^−1^. Fine-scale valley networks in this area are mostly pitted types (~72%) with low Strahler stream orders (1–2). The eastern exposure, downgradient of the southern exposure, has the most mapped valley networks among the three with ~78% pitted types, dendritic branching style networks, higher Strahler stream orders (1–4), and a network density of 0.042 km^−1^. The apparent source regions of many valley networks can be traced to topographic scarps at the contacts between etched and ancient cratered terrains. This may indicate a combination of localized and regional fluvial sources. Further downgradient, the western exposure has mostly single-thread ridge types (61%) with little branching, low stream orders (1–2), and a network density of 0.015 km^−1^. In summary, the overall trend is from dendritic, high order pitted and(or) classical style valley networks in the south to single thread, low order ridge types down to the west.

Of the remaining mapped fine-scale valley networks (~33%), they are mostly within *Nhc2* and etched units that are adjacent to the three regional exposures of *Nhc1*. Valley networks in *Nhc2* are ~74% pitted types, consistent with the eastern *Nhc1* exposure and the nearly coeval formation of these two units. Etched units *NMe1*, *NMe2*, and *HNMe3* have ~60% ridge type valley networks that can be as high as ~68% if transitional types with at least one ridge type are included. The Strahler stream order, including transitional types, are dominantly order one (~88%) which suggests that single-threaded ridges (formerly single channel flows) continued from the end of regional transitions in ancient cratered terrain into younger layered sedimentary deposits.

Based on the stratigraphy of geologic units, unit ages derived from previous studies, morphologic characteristics of valley networks, and potential terrestrial analogs for the mapped features, we hypothesize the following sequence of events in the map region. The earliest phase of valley network formation involved sustained overland flow, aqueous erosion of the surface, and the formation of classic valley networks. This was followed by a period of dominant groundwater processes leading to widespread evaporite deposition. Later dissolution of solutes from fluctuating groundwater levels reactivated some of the former valley networks (mostly in eastern *Nhc1* and parts of *Nhc2*). The dissolution and subsequent collapse of the surface is reflected by the formation of pitted valley networks. The pitted types represent the most recent record of aqueous activity that are now visible due to substantial erosion of the GMP region. Overall, our mapping results and interpretation of events are consistent with episodic aqueous activity rather than a monotonic decline in climate conditions associated with the transition from the Noachian to Hesperian period.

## Supplementary Material

Map
