# Supplementary material for: Valley network morphology in the greater Meridiani Planum region, Mars
Source: J Maps. Author manuscript; Available in PMC 2021 Oct 27. (PMC8549781; doi:10.1080/17445647.2018.1530154)
Supplement: Map [file NIHMS1746535-supplement-Map.pdf]

Valley Network Morphology in the Greater Meridiani Planum Region, Mars

Frank C. Chuang and Rebecca M. E. Williams

Planetary Science Institute, 1700 E Fort Lowell Rd., Suite 106, Tucson, AZ 85719 USA (e-mail: chuang@psi.edu)

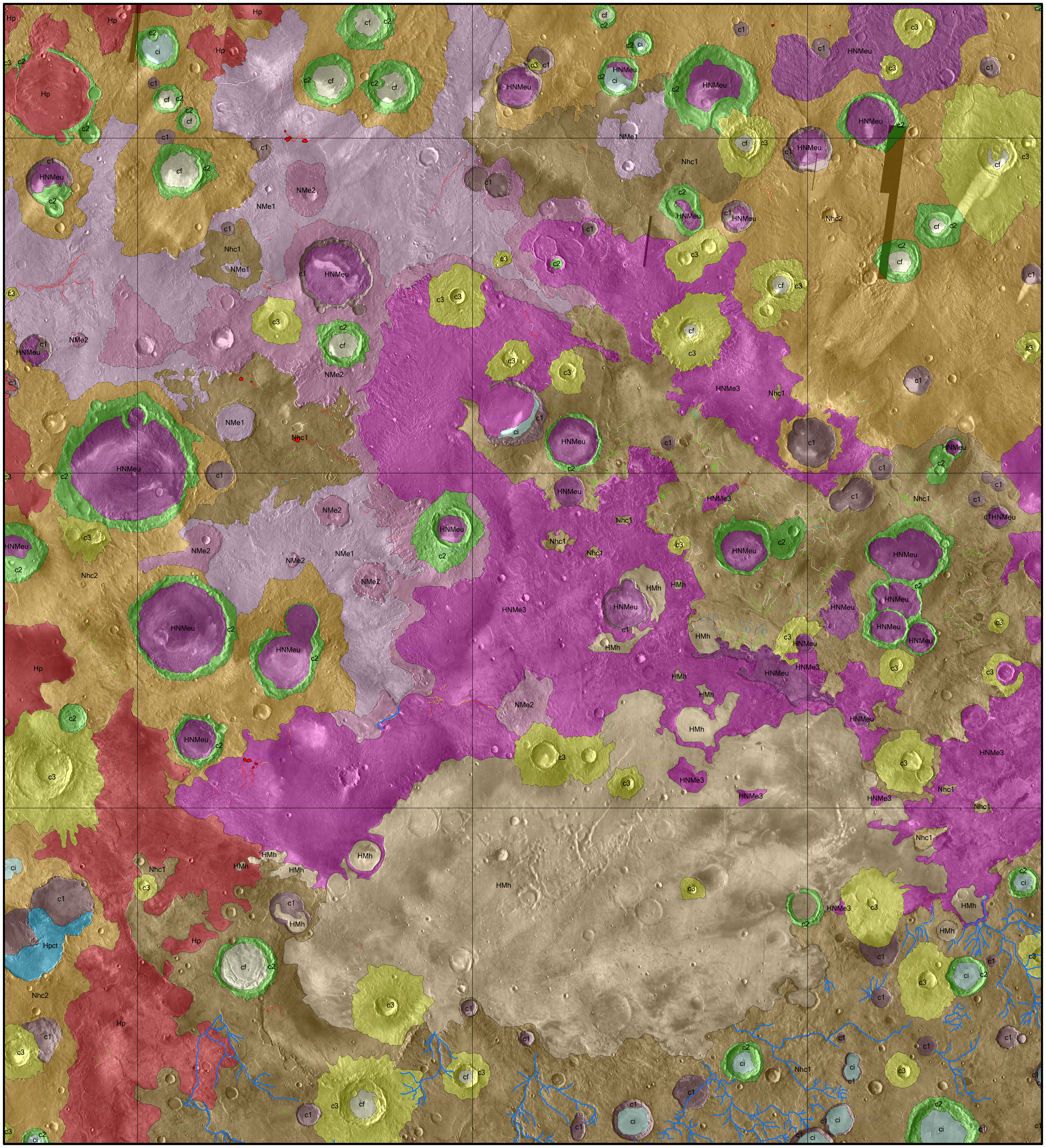

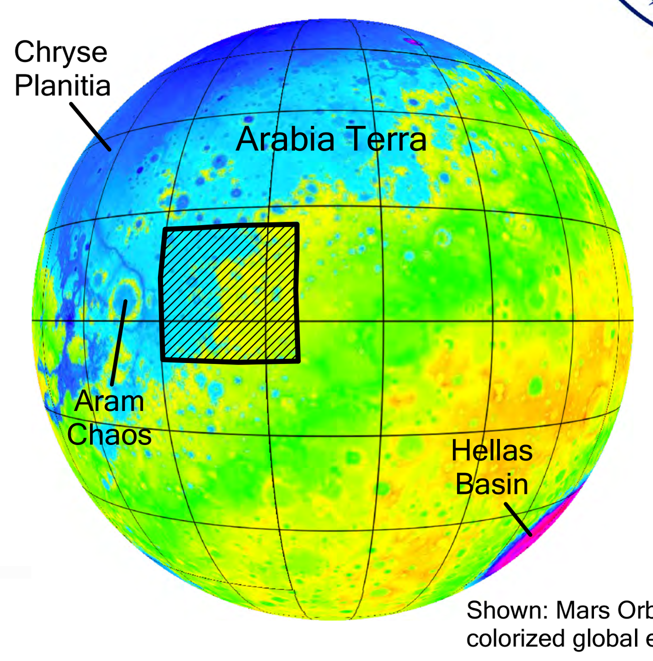

Shown: Mars Orbiter Laser Altimeter (MOLA) colorized global elevation data (463 m/pixel)

N

LEGEND

Valley Network Morphologic Types

- Ridges (Ridge1-Ridge7)
- Troughs (Trough1-Trough2)
- Channels
- Pitted
- Knobs
- Two or more types
- Classic Valley Network
- Candidate Paleolakes
- Hynek et al. (2010) Valley Networks

Hynek and Di Achille (2017)

Geologic Units (In stratigraphic order (oldest at bottom))

- Chaotic (Hpct)
- Plains (Hp)
- Hematite (HMh)
- Undivided etched (HNMeu)
- Upper etched (HNMe3)
- Middle etched (NMe2)
- Lower etched (NMe1)
- Cratered (Nhc2)
- Subdued crater (Nhc1)
- Internal crater deposits (ci)
- Crater fill (cf)
- Well-preserved crater (c3)
- Moderately eroded crater (c2)
- Highly eroded crater (c1)

Geologic Contacts

- Approximate
- Certain

Scale 1:1,000,000

0 100 200 300 400 500 **Kilometers**

Geologic units from Hynek and Di Achille (2017) draped over THEMIS daytime IR global mosaic (100 m/pixel)

Map projection: Equidistant Cylindrical, Geoid: IAU Mars 2000 Sphere (3396190 m semimajor and semiminor axes), Central Meridian and Standard Parallel: 0

REFERENCES

Hynek, B. M., & Di Achille, G. (2017). Geologic Map of Meridiani Planum, Mars. U. S. Geological Survey Scientific Investigations Map 3356. Scale 1:20,000,000. https://doi.org/10.3133/sim3356.

Hynek, B. M., Beach, M., & Hoke, M. R. T. (2010). Updated global map of Martian valley networks and implications for climate and hydrologic processes. Journal of Geophysical Research, 115. doi:10.1029/2009JE003548.

© Journal of Maps, 2018
